# Adaptive trial designs: a review of barriers and opportunities

**DOI:** 10.1186/1745-6215-13-145

**Published:** 2012-08-23

**Authors:** John A Kairalla, Christopher S Coffey, Mitchell A Thomann, Keith E Muller

**Affiliations:** 1 Department of Biostatistics, University of Florida, PO Box 117450, Gainesville, FL, 32611-7450, USA; 2 Department of Biostatistics, University of Iowa, 2400 University Capitol Centre, Iowa City, IA, 52240-4034, USA; 3 Department of Health Outcomes and Policy, University of Florida, PO Box 100177, Gainesville, FL, 32610-0177, USA

**Keywords:** Adaptive designs, Flexible designs, Group sequential, Internal pilot, Power, Sample size re-estimation, Comparative effectiveness research, Small clinical trials

## Abstract

Adaptive designs allow planned modifications based on data accumulating within a study. The promise of greater flexibility and efficiency stimulates increasing interest in adaptive designs from clinical, academic, and regulatory parties. When adaptive designs are used properly, efficiencies can include a smaller sample size, a more efficient treatment development process, and an increased chance of correctly answering the clinical question of interest. However, improper adaptations can lead to biased studies. A broad definition of adaptive designs allows for countless variations, which creates confusion as to the statistical validity and practical feasibility of many designs. Determining properties of a particular adaptive design requires careful consideration of the scientific context and statistical assumptions. We first review several adaptive designs that garner the most current interest. We focus on the design principles and research issues that lead to particular designs being appealing or unappealing in particular applications. We separately discuss exploratory and confirmatory stage designs in order to account for the differences in regulatory concerns. We include adaptive seamless designs, which combine stages in a unified approach. We also highlight a number of applied areas, such as comparative effectiveness research, that would benefit from the use of adaptive designs. Finally, we describe a number of current barriers and provide initial suggestions for overcoming them in order to promote wider use of appropriate adaptive designs. Given the breadth of the coverage all mathematical and most implementation details are omitted for the sake of brevity. However, the interested reader will find that we provide current references to focused reviews and original theoretical sources which lead to details of the current state of the art in theory and practice.

## Review

### Introduction

In traditional clinical trials, key elements such as primary endpoint, clinically meaningful treatment difference, and measure of variability are pre-specified during planning in order to design the study. Investigators then collect all data and perform analyses. The success of the study depends on the accuracy of the original assumptions. Adaptive Designs (ADs) give one way to address uncertainty about choices made during planning. ADs allow a review of accumulating information during a trial to possibly modify trial characteristics [1]. The flexibility can translate into more efficient therapy development by reducing trial size. The flexibility also increases the chance of a ‘successful’ trial that answers the question of interest (finding a significant effect if one exists or stopping the trial as early as possible if no effect exists).

ADs have received a great deal of attention in the statistical, pharmaceutical, and regulatory fields [1-8]. The rapid proliferation of interest and inconsistent use of terminology has created confusion and controversy about similarities and differences among the various techniques. Even the definition of an ‘adaptive design’ is a source of confusion. Fortunately, two recent publications have reduced the confusion. An AD working group was formed in 2005 in order to ‘foster and facilitate wider usage and regulatory acceptance of ADs and to enhance clinical development, through fact-based evaluation of the benefits and challenges associated with these designs’ [2]. The group was originally sponsored by the Pharmaceutical Research and Manufacturers of America (PhRMA) and is currently sponsored by the Drug Information Association. The group defined an AD as ‘a clinical study design that uses accumulating data to decide how to modify aspects of the study as it continues, without undermining the validity and integrity of the trial.’ The group also stressed that the changes should not be *ad hoc*, but ‘by design.’ Finally, the group emphasized that ADs are not a solution for inadequate planning, but are meant to enhance study efficiency while maintaining validity and integrity. Subsequently, the US Food and Drug Administration (FDA) released a draft version of the “Guidance for Industry: Adaptive Design Clinical Trials for Drugs and Biologics” [3]. The document defined an AD as ‘a study that includes a prospectively planned opportunity for modification of one or more specified aspects of the study design and hypotheses based on analysis of data (usually interim data) from subjects in the study.’ Both groups supported the notion that changes are based on pre-specified decision rules. However, the FDA defined ADs more generally by interpreting as ‘prospective’ any adaptations planned ‘before data were examined in an unblinded manner by any personnel involved in planning the revision’ [3]. Since different individuals become unblinded (that is, ‘unmasked’) at different points in a trial, we believe the FDA draft guidance document left open doors to some gray areas that merit further discussion. Both groups made it clear that the most valid ADs follow the principle of ‘adaptive by design’ since that is the only way to ensure that the integrity and validity of the trial are not compromised by the adaptations.

**Figure 1:**
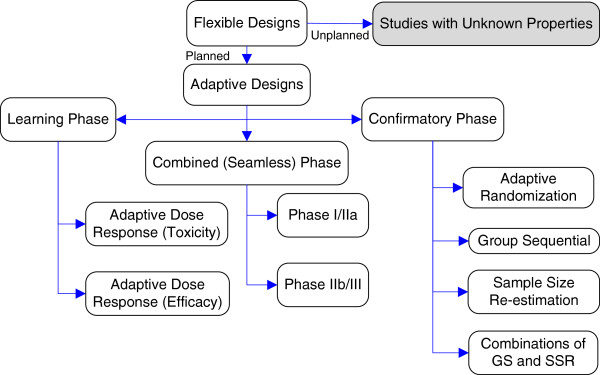
Summary of different types of adaptive designs for clinical trials.

It is important to differentiate between ADs and what others have referred to as flexible designs [1,9]. The difference was perhaps best described by Brannath *et al.* who state, that ‘Many designs have been suggested which incorporate adaptivity, however, are in no means flexible, since the rule of how the interim data determine the design of the second part of the trial is assumed to be completely specified in advance’ [9]. Thus, a flexible design describes a more general type of study design that incorporates both planned and unplanned features (Figure 1). There is general agreement that the implementation of flexible designs cannot be haphazard but must preserve validity and integrity (for example, by controlling type I error rate). While attractive, we believe that this flexibility opens a trial to potential criticism from outside observers and regulators. Furthermore, we believe that many of the concerns could be eliminated by giving more thought to potential adaptations during the planning stages of a trial. Correspondingly, for this review, we adopt a definition similar to that of the AD working group and of the FDA and focus only on ADs that use information from within-trial accumulating data to make changes based on preplanned rules. 

As Figure 1 demonstrates, even the constrained definition of AD allows a wide range of possible adaptations, some more acceptable than others. The designs allow updates to the maximum sample size, study duration, treatment group allocation, dosing, number of treatment arms, or study endpoints. For each type of adaptation, researchers must ensure that the type I error rate is controlled, the trial has a high probability of answering the research question of interest, and equipoise is maintained [10]. New analytic results with properly designed simulations [11] are often needed to meet the restrictions. The approach reinforces the importance of ‘adaptive by design’ because the adaptation rules must be clearly specified in advance in order to properly design the simulations.

Despite their suggested promise, current acceptance and use of ADs in clinical trials are not aligned with the attention given to ADs in the literature. In order to justify the use of ADs, more work is needed to clarify which designs are appropriate, and what needs to be done to ensure successful implementation. In the remainder of the paper we summarize specific AD types used in clinical research and address current concerns with the use of the designs. There are too many possible ADs to cover all of them in a brief review. We begin with learning stage designs. Next, we describe confirmatory stage designs. We then discuss adaptive seamless designs that seek to integrate multiple stages of clinical research into a single study. Next we explore applied areas that would benefit from ADs. Finally, we describe some barriers to the implementation of ADs and suggest how they can be resolved in order to make appropriate ADs practical.

### Learning-stage adaptive designs

#### Overview

In general, AD methods are accepted more in the learning (exploratory) stages of clinical trials [3,4]. Early in the clinical development process ADs allow researchers to learn and optimize based on accruing information related to dosing, exposure, differential participant response, response modifiers, or biomarker responses [3]. The low impact of exploratory studies on regulatory approval means less emphasis on control of type I errors, and more emphasis on control of type II errors (avoiding false negatives). Early learning phase designs in areas with potentially toxic treatments (for example, cancer or some neurological diseases) seek to determine the maximum tolerated dose (MTD), the highest dose for less than some percent of treated participants (such as 33 or 50 percent) having dose-related toxicities. An accurate determination of the MTD is critical since it will likely be used as the maximum dose in future clinical development. If the dose is too low, a potentially useful drug could be missed. If the dose is too high, participants in future studies could be put at risk. After the MTD has been determined, the next step is typically to choose a dose (less than or equal to the MTD) most likely to affect the clinical outcome of interest. Since the issues are very different for these two phases of the learning stage, we briefly summarize each below.

#### Early learning stage (toxicity dose)

Although a number of methods have been proposed for phase I MTD determination, by far the most prevalent is the traditional 3 + 3 method originally developed for, and primarily used in, oncology trials [12,13]. In this rule-based method, toxicity is defined as a binary event and participants are treated in groups of three, starting with an initial low dose. The algorithm then iterates, moving dose levels up or down depending on the number of toxicities observed. The MTD is identified from the data; for example, the highest dose studied with less than 1/3 toxicities (that is, zero or one dose-limiting toxicity out of six participants). This method is straightforward and convenient in that it requires no modeling and very few assumptions. However, the method has been criticized for not producing a good estimate [14]. Several adaptive dose-response methods have advantages over the traditional method. A popular design is the Bayesian adaptive model-based approach called the continual reassessment method (CRM) [14]. By more effectively estimating the MTD along with a dose-response curve, the CRM tends to quickly accelerate participants to doses around the MTD. Fewer participants are treated at ineffective doses and the design is less likely to over-estimate or under-estimate the true MTD compared to the 3 + 3 method [14]. Safety concerns about the original CRM led to several improvements [15,16]. The CRM has utility in any area where finding the MTD is needed. However, to date, it has primarily been used in cancer [17] and stroke [18,19] research trials.

#### Late learning stage exploratory (efficacy dose)

ADs for later exploratory development are not as well-developed as for earlier work. Consequently, PhRMA created a separate adaptive dose response working group to explore the issue and make recommendations [20]. Among the group’s conclusions were that dose response (DR) is more easily detected than estimated, typical sample sizes in dose-ranging studies are inadequate for DR estimation, and adaptive dose-ranging methods clearly improve DR detection and estimation. The group also noted the advantages of design-focused adaptive methods. The group favored a general adaptive dose allocation approach using Bayesian modeling to identify an appropriate dose for each new participant based on previous responses [21], as employed in the Acute Stroke Therapy by Inhibition of Neutrophils (ASTIN) study [22]. Unfortunately, complex simulations (or new analytic development) and software are needed in order to control the operating characteristics and employ the methods. The development of well documented and user-friendly software is vital for future use. We believe that access to dependable and easy-to-use software will make ADs more common in the exploratory stages of trials.

### Confirmatory adaptive designs

#### Overview

From the FDA’s current perspective, some designs are considered ‘well understood,’ while others are not [3]. Accordingly, scrutiny of a protocol will vary depending on the type of design proposed. The FDA generally accepts study designs that base adaptations on masked (aggregate) data [3]. For example, a study could change recruitment criteria based on accruing aggregate baseline measurements. Group sequential (GS) designs are also deemed ‘well understood’ by the FDA. GS designs allow stopping a trial early if it becomes clear that a treatment is superior or inferior. Thus, GS methods meet our definition of an AD and are by far the most widely used ADs in modern confirmatory clinical research. They have been extensively described elsewhere [23] and will not be discussed further.

Some designs are ‘less well understood,’ from the FDA perspective [3]. It is important to note that such methods are not automatically prohibited by the FDA. Rather, there is a higher bar for justifying the use of less well-understood designs. Proving lack of bias and advantageous operating characteristics requires extensive planning and validation. Debate continues concerning the usefulness and validity of confirmatory ADs in the category. Examples include adaptive randomization, enrichment designs, and sample size re-estimation (although some subtypes are classified as ‘well understood’). We briefly mention each below.

#### Adaptive randomization

Traditional randomization fixes constant allocation probabilities in advance. Adaptive randomization methods vary the allocation of subjects to treatment groups based on accruing trial information [1,24,25]. There are two basic types: covariate and response adaptive randomization. Each is briefly described immediately below.

With a sufficient sample size, a traditional randomization process will balance the distribution of all known and unknown covariates at the end of a study. This is, in fact, one of the major benefits of randomization. However, this process does not ensure that the covariates are balanced at all times during the conduct of the trial. *Covariate adaptive randomization* provides a higher probability of having treatment group balanced covariates during the study by allowing the allocation probabilities to change as a function of the current distribution of covariates. Methods exist forcing optimum balance deterministically (for example, minimization), with fixed (unequal) probability, and with dynamic allocation probabilities [26]. A number of examples of methods and practice can be found in the literature (for example, [27,28]).

Alternatively, *response adaptive randomization* uses observed treatment outcomes from preceding participants to change allocation probabilities. The strategy can fulfill the ethical desire to increase the likelihood of giving an individual the best-known treatment at the time of randomization. Use is not widespread, but examples can be found [29-32]. Although attractive, response adaptive randomization schemes have administrative complexities and may create ethical dilemmas [7,33]. One complication is that enrolling later in the study increases the chance of receiving the superior treatment since the randomization probability will have increased for the better treatment. Thus, bias can be created if sicker patients enroll earlier and healthier ones decide to wait until later to enroll [5]. Furthermore, the actual advantages may be negligible since the analysis, type I error rate control, and sample size calculations become more complicated due to the need to account for adaptive randomization [34-36]. Proponents of response-adaptive randomization designs defend their efficiency and usefulness while continuing to address criticisms with new methods and simulation results [25]. However, according to the FDA draft guidance, ‘Adaptive randomization should be used cautiously in adequate and well-controlled studies, as the analysis is not as easily interpretable as when fixed randomization probabilities are used’ [3].

#### Enrichment designs

Enrichment of a study population refers to ensuring that participants in a trial are likely to demonstrate an effect from treatment, if one exists [37]. For example, there is benefit to enrolling participants lacking comorbidities, with a risk factor of interest (such as high blood pressure), and likely to be compliant. An extension known as adaptive enrichment designs fulfills the desire to target therapies to patients who can benefit the most from the treatment [38,39]. In such designs, a trial initially considers a broad population. The first study period reveals participant groups most likely to benefit from the test agent (discovery phase). Subgroup members are then randomized to receive either the active agent or control (validation phase). Power for the chosen subgroups is increased due to the increased sample size in the subgroups, while non-promising groups are discarded. Adaptive enrichment designs have been praised for their ability to identify patient groups and undiluted effect sizes that can aid in the design and efficiency of replication studies [39]. An appealing area for adaptive enrichment is pharmacogenetic research where it could allow for isolation of the one or two genetic marker subgroups that are predictive for treatment response. The approach can increase efficiency when identifiable genetic subgroups have increased treatment benefit [40]. Additionally, some studies have used an adaptive enrichment to identify a subset most likely to respond to treatment [41]. However, adaptive enrichment designs have been criticized as having unfavorable operating characteristics in real-world confirmatory research. Disadvantages include increases in complexity, biased treatment effect estimates, lack of generalizability, and lack of information in excluded groups [7]. We believe that adaptive enrichment designs currently have greatest value in late learning stage designs.

#### Sample size re-estimation

Choosing a fixed sample size is complicated by the need to choose a clinically meaningful treatment effect and to specify values for nuisance parameters such as the variance, overall event rate, or accrual rate. Inaccurate estimates of the parameters lead to an underpowered or overpowered study, both of which have negative consequences. Sample size re-estimation (SSR) designs allow the parameter estimates to be updated during an ongoing trial, and then used to adjust the sample size accordingly [42].

Historically, a great deal of controversy surrounding ADs has centered on SSR based on observed treatment effects [43-45]. The methods are defended for use in specific contexts, such as using a small amount of initial funding to seek promising results [46]. The authors of the FDA draft guidance document, in listing the design as ‘less well understood,’ noted the potential for inefficiency, an increased type I error rate, difficulties in interpretation, and magnification of treatment effect bias [3]. A major concern with this type of SSR design is the potential to convey treatment effect information from decisions made using treatment-arm specific data at interim time points. A clever investigator with knowledge of the SSR procedure and the decision made after viewing the data could possibly back-calculate an absolute treatment effect. It should be noted that concerns of gaining some knowledge based on an action (or inaction) exist when using any treatment-arm specific data, including GS methods. Nevertheless, the clinical trials community now routinely uses GS methods without major concerns since the conveyed information is usually minimal.

Other types of SSR have stimulated less controversy. For example, internal pilots (IPs) are two stage designs with no interim testing, but with interim SSR based only on first stage nuisance parameter estimates [47]. Moderate to large sample sizes imply minimal type I error rate inflation with unadjusted tests in a range of settings [4,48,49]. IP designs can be used in large randomized controlled trials to re-assess key nuisance parameters and make appropriate modifications with little cost to type I error rate. In contrast, small IP trials can have inflated type I error rate and therefore require adjustments for bias [50-52]. Since IP designs do not include interim testing or effect size based SSR, there generally are not the same concerns about indirectly conveying an absolute treatment effect, though Proschan showed that it is possible if a researcher has knowledge of both the IP procedure and access to the blinded data [48]. Consequently, some observers believe that, from a regulatory standpoint, IP methods that keep group allocation masked may be preferred whenever possible. Accordingly, masked methods for IPs have been proposed [53,54] and are classified as ‘well understood’ in the FDA Draft Guidance document [3]. However, unmasked IP procedures may be appropriate provided that steps are taken to minimize the number of people with access to data or to the group allocation. Whether blinded or not, if an IP design is implemented in a setting where non-objective parties do not have access to accumulating raw data, the sample size changes will give no information concerning effect trends of interest. Thus, we believe that the setting has fewer risks and therefore encourage more use of SSR based on nuisance parameters in future phase II and III trials.

#### Adaptive seamless designs

A seamless design combines exploratory and confirmatory phases into a single trial. As a type of two-stage design, seamless designs can increase overall efficiency by reducing the lead time (‘white space’) between phases. Information from participants enrolled in the first stage is used to inform the second stage. An *adaptive seamless design* proceeds in the same manner, but uses data from participants enrolled in both stages in the final analysis. Previous authors have paid the most attention to a seamless transition between phase IIb (learning) and phase III (confirming) [1,55-58]. Seamless designs also seem appealing in early development (phase I/IIa). The approach allows for a more efficient utilization of sample size and resources versus conducting completely separate studies. However, since data from the learning phase inform decisions for the second phase, using the data in the final analysis raises concerns about bias and error rate inflation. As an example, consider the Coenzyme Q10 in Amyotrophic Lateral Sclerosis (QALS) study: an adaptive, two-stage, randomized controlled phase I/IIa trial to compare decline in Amyotrophic Lateral Sclerosis (ALS) Functional Rating Scale score [59]. The first phase used a selection design [60] to choose one of two doses (1800 mg or 2500 mg). The second phase then compared the selected dose to placebo using a futility design [61]. Because the second phase dose was selected as ‘best’ in the first phase, there is a positive bias carried forward. Correspondingly, if the final test does not account for the bias, the overall type I error rate may be increased. The QALS investigators performed a series of studies to determine a bias correction and incorporated it into the final test statistic [62]. The scenario is common since seamless designs require special statistical methods and extra planning to account for the potential bias. In general, the potential benefits must be weighed against the additional effort required to ensure a valid test at the end of the study.

### Applied areas that would benefit from adaptive designs

#### Combinations of group sequential and sample size re-estimation

Combining the power benefits of an IP design and the early stopping sample size advantages of GS designs has great appeal. Asymptotically correct information-based monitoring approaches for simultaneous use of GS and IP methods in large clinical trials have been proposed [63]. The approach can give power and expected sample size benefits over fixed sample methods in small samples, but may inflate the type I error rate [64]. Kairalla *et al.*[65] provided a practical solution; however, more work is needed in the area.

#### Rare diseases and small trials

Planning a small clinical trial, particularly for a rare disease, presents several challenges. Any trial should examine an important research question, use a rigorous and sensitive methodology to address the question, and minimize risks to participants. Choosing a feasible study design to accomplish all of the goals in a small trial can be a formidable challenge. Small trials exhibit more variability than larger trials, which implies that standard designs may lead to trials with power adequate only for large effects. The setting makes ADs particularly appealing. However, it is important to be clear about what an AD can and cannot do in the rare disease setting. Most importantly, an AD cannot make a drug more effective. One of the biggest benefits of an AD is quite the opposite: identifying ineffective treatments earlier. Doing so will minimize the resources allocated to studying an ineffective treatment and allow re-distributing resources to more promising treatments. Although ADs cannot ‘change the answer’ regarding the effectiveness of a particular treatment, they can increase the efficiency in finding an answer.

#### Comparative effectiveness trials

Comparative effectiveness (CE) trials compare two or more treatments [66] that have already been shown to be efficacious. Unique issues found in CE trials make ADs attractive in the area. For one, the concept of a ‘minimum clinically meaningful effect’ in the population has a diminished meaning in a CE trial. Assuming roughly equal costs and side effects, a range of values may be identified with upper limit the largest reasonable effect and lower limit the smallest effect deemed sizable enough to change practice in the study context. Unfortunately, since detecting smaller effects requires larger sample sizes, for practical reasons researchers may feel the need to power CE trials for effects on the upper end of the spectrum. A potential AD could have two stages with the first powered to detect the larger reasonable effect size. At the conclusion of the first stage, one of three decisions might be reached: 1) Declare efficacy (one treatment best); 2) Declare futility (unlikely to show difference between treatments); or 3) If evidence suggests a smaller effect might exist, then proceed with a second stage powered to detect the smaller effect. Another issue is that available variability estimates are probably too low since the estimates were likely obtained from highly controlled efficacy trials. If true, using the estimates to power a CE trial may lead to an underpowered study. Thus, variance-based SSR could be built into the prior example to address the uncertainty. We believe ADs have promise in CE trials and that future research is warranted.

#### Applications in other research settings

Currently, ADs are considered most often in the context of clinical trials. However, the ability to modify incorrect initial assumptions would have value in many other settings. Importantly, since regulatory issues may not exist in many research settings, we believe that ADs may actually be much easier to implement. For example, laboratory research involving animals could use an AD to re-assess key parameters and determine whether more animals are needed to achieve high power. As another example, an observational study requires assumptions about the distribution of the population that will be enrolled. Any discrepancy between the hypothesized and actual distribution of the enrolled population will affect the power of the study. Although extensions of the IP design to the observational setting have been considered [67], more work is needed.

### Barriers to implementing adaptive designs

Even though additional methodological development is needed in ADs, appropriate statistical methods exist to support a much greater use of ADs than currently seen. We believe logistical issues and regulatory concerns, rather than statistical issues, currently limit AD use. The majority of research on ADs has been driven by drug development within the pharmaceutical industry. While many basic principles remain the same regardless of the funding environment, some specific challenges differ when considering the use of ADs for trials funded by the National Institutes of Health (NIH) or foundations. For example, traditional funding mechanisms lack the required flexibility to account for sample size modifications after initiation of a trial. There is also a general sense of confusion and lack of understanding about the distinction between acceptable and unacceptable adaptations. If the reviewers do not understand the important distinctions, a valid AD might not pass through peer review. An NIH and private foundation funded workshop on ‘Scientific Advances in Adaptive Clinical Trial Designs’ was held in November 2009, as a first attempt to address the challenges [68]. Participants included representatives from research institutions, regulatory bodies, patient advocacy groups, non-profit organizations, professional associations, and pharmaceutical companies. The participants stressed that the use of ADs may require a different way of thinking about the structure and conduct of Data and Safety Monitoring Boards (DSMBs). Also, they agreed that there is a great need for further education and communication regarding the strengths and weaknesses of various types of ADs. For example, researchers should be encouraged to publish manuscripts describing experiences (both positive and negative) associated with completed trials that used an AD. Similarly, a stronger emphasis on a statistical background for NIH reviewers and DSMB members seems necessary.

While communication among parties can go a long way towards increasing the use and understanding of ADs, more work is needed to develop infrastructure to support AD trials. Study infrastructure is one area where industry is clearly ahead of grant funded research. As an example, justifying properties of ADs often requires extensive planning through computations or simulations. Researchers must find a way to fund the creation of extensive calculations for a hypothetical study. The issue is exacerbated by fact that the planning is generally required prior to submitting a grant application for funding. Many pharmaceutical companies are developing in-house teams primarily responsible for conducting such simulations. Greater barriers exist for implementing the same type of infrastructure within publicly funded environments, particularly given the challenges associated with the current limited and highly competitive federal budget.

In our opinion, the most important way to ensure a high chance of conducting a successful AD trial is to have a high level of infrastructure (efficient data management, thorough understanding of AD issues, *etcetera*) in place. A low complexity AD (for example, an IP or GS design) conducted in a high infrastructure environment currently provides the best chance for success. However, a low infrastructure environment might be able to successfully conduct a low complexity AD, with a little bit of extra effort. The same chance of success is not present if one is trying to implement a high complexity AD design (for example, an adaptive seamless II/III design, or a combination of different adaptations). With a complex design a high level of infrastructure is needed in order to successfully conduct the trial. The QALS study, a complex two-stage seamless design described earlier, is a good example of a study with high infrastructure and with high adaptivity [62]. The QALS study was a success, requiring only 185 participants to establish that the cost and effort of undertaking a phase III trial would not be worthwhile. However, the trial was successful only because all parties involved (researchers, sponsor, DSMB members, *etcetera*) clearly understood the intricacies of the AD being used. A break-down in understanding for any stakeholder could have severely damaged the study. A high complexity AD with low infrastructure is likely doomed to fail. Unfortunately, the scenario is currently a common one due to the desire to use complex adaptive designs without the necessary high level of infrastructure required for success. One solution would be to only consider simple ADs. However, since researchers are mainly interested in obtaining the efficiency and advantages of more complex adaptations, we believe that the only way to increase the chances for success in the future is to first improve the existing infrastructure. As previously stated, many companies have begun the process. However, we believe that NIH should also offer more recognition and funding for planning clinical trials that might benefit from adaptations.

Although infrastructure characteristics often limit rates of adaptation, a number of steps have been taken to address the concern, especially in the neurosciences. One ongoing example is the NIH and FDA supported ‘Accelerating Drug and Device Evaluation through Innovative Clinical Trial Design’ project [69]. The participants are studying the development and acceptance of a wide range of adaptive designs within the existing infrastructure of the National Institute of Neurological Disorders and Stroke (NINDS)-supported Neurological Emergencies Treatment Trials (NETT) network [70]. The goal is to incorporate the resulting designs into future network grant submissions. Another example is the creation of the NINDS-funded Network for Excellence in Neuroscience Clinical Trials (NeuroNEXT) [71]. The goal of the network is to provide infrastructure supporting phase II studies in neuroscience, including the conduct of studies in rare neurological diseases. The long-term objective of the network is to rapidly and efficiently translate advances in neuroscience into treatments for individuals with neurologic disorders. The infrastructure is intended to serve as a model that can be replicated across a number of studies and diseases. The development of rich infrastructures such as NeuroNEXT greatly increases the feasibility of using more novel trial designs, including ADs. Additional infrastructure with flexibility is needed in other disease areas to advance the use of ADs, particularly in the publicly funded environment.

## Conclusions

A general overview of the main design classes provides the basis for discussing how to correctly implement ADs. We agree with Vandemeulebroecke [72] that discussion concerning ADs should center on five main points: feasibility, validity, integrity, efficiency, and flexibility. We recommend systematically addressing each of the concerns through the development of better methodology, infrastructure, and software. Successful adoption of ADs also requires systematic changes to clinical research policies. We believe that the barriers can be overcome to move appropriate ADs into common clinical practice.

## Abbreviations

AD: Adaptive designs; ALS: Amyotrophic Lateral Sclerosis; ASTIN: Acute Stroke Therapy by Inhibition of Neutrophils; CE: Comparative effectiveness; CRM: Continual reassessment method; DSMB: Data and Safety Monitoring Board; DR: Dose response; FDA: US Food and Drug Administration; GS: Group sequential; IP: Internal pilot; MTD: Maximum tolerated dose; NETT: Neurological Emergencies Treatment Trials; NeuroNEXT: Network for Excellence in Neuroscience Clinical Trials; NIH: National Institutes of Health; NINDS: National Institute of Neurological Disorders and Stroke; PhRMA: Pharmaceutical Research and Manufacturers of America; QALS: Coenzyme Q10 in ALS; SSR: Sample size re-estimation.

## Competing interests

The authors declare that they have no competing interests.

## Authors’ contributions

All authors contributed significantly to the overall design of the paper. JAK wrote the initial draft and worked on revisions. CSC conceived of the paper and worked on revisions. MAT conducted literature reviews and worked on revisions. KEM contributed to the overall focus and content and helped revise the manuscript. All authors read and approved the final manuscript.

## References

[B1] Chow S Chang M Adaptive design methods in clinical trials 2007 Boca Raton: Chapman & Hall/CRC

[B2] Gallo P Chuang-Stein C Dragalin V Gaydos B Krams M Pinheiro J Adaptive designs in clinical drug development: an executive summary of the PhRMA working group J Biopharm Stat 2006 16 275 283 10.1080/10543400600614742 16724485

[B3] U.S. Food and Drug Administration Draft Guidance for Industry: adaptive design clinical trials for drugs and biologics http://www.fda.gov/downloads/DrugsGuidanceComplianceRegulatoryInformation/Guidances/UCM201790.pdf 10.1080/10543406.2010.514453 21058108

[B4] Coffey CS Kairalla JA Adaptive clinical trials: progress and challenges Drugs R&D 2008 9 229 242 10.2165/00126839-200809040-00003 18588354

[B5] Chow SC Corey R Benefits, challenges and obstacles of adaptive clinical trial designs Orph J Rare Dis 2011 6 79 10.1186/1750-1172-6-79 PMC3248853 22129361

[B6] Bretz F Koenig F Brannath W Glimm E Posch M Adaptive designs for confirmatory clinical trials Stat Med 2009 28 1181 1217 10.1002/sim.3538 19206095

[B7] Emerson SS Fleming TR Adaptive Methods: Telling “The Rest of the Story” J Biopharm Stat 2010 20 1150 1165 10.1080/10543406.2010.514457 21058111

[B8] Coffey CS Ravina B, Cummings J, McDermott M, Poole RM Adaptive Design Across Stages of Therapeutic Development Clinical Trials in Neurology: Design, Conduct, & Analysis 2012 Cambridge: Cambridge University Press 91 100

[B9] Brannath W Koenig F Bauer P Multiplicity and flexibility in clinical trials Pharm Stat 2007 6 205 216 10.1002/pst.302 17674349

[B10] Dragalin V Adaptive designs: terminology and classification Drug Inf J 2006 40 425 435

[B11] Burton A Altman DG Royston P Holder RL The design of simulation studies in medical statistics Stat Med 2006 24 4279 4292 16947139 10.1002/sim.2673

[B12] Storer BE Design and Analysis of Phase I Clinical Trials Biometrics 1989 45 925 937 10.2307/2531693 2790129

[B13] Tourneau CL Lee JJ Siu LL Dose Escalation Methods in Phase I Cancer Clinical Trials J Natl Cancer I 2009 101 708 720 10.1093/jnci/djp079 PMC2684552 19436029

[B14] O’Quigley J Pepe M Fisher L Continual reassessment method: a practical design for phase I clinical trials in cancer Biometrics 1990 46 33 48 10.2307/2531628 2350571

[B15] Cheung K Kaufmann P Efficiency perspectives on adaptive designs in stroke clinical trials Stroke 2011 42 2990 2994 10.1161/STROKEAHA.111.620765 21885845 PMC3183258

[B16] Garrett-Mayer E The continual reassessment method for dose-finding studies: a tutorial Clin Trials 2006 3 57 71 10.1191/1740774506cn134oa 16539090

[B17] Tevaarwerk A Wilding G Eickhoff J Chappell R Sidor C Arnott J Bailey H Schelman W Liu G Phase I study of continuous MKC-1 in patients with advanced or metastatic solid malignancies using the modified Time-to-Event Continual Reassessment Method (TITE-CRM) dose escalation design Invest New Drugs 2011 30 1039 1045 21225315 10.1007/s10637-010-9629-6 PMC3139017

[B18] Elkind MSV Sacco RL MacArthur RB Peerschke E Neils G Andrews H Stillman J Corporan T Leifer D Liu R Cheung K High-dose Lovastatin for acute ischemic stroke: Results of the phase I dose escalation neuroprotection with statin therapy for acute recovery trial (NeuSTART) Cerebrovasc Dis 2009 28 266 275 10.1159/000228709 19609078 PMC2814015

[B19] Selim M Yeatts S Goldstein JN Gomes J Greenberg S Morgenstern LB Schlaug G Torbey M Waldman B Xi G Palesch Y Safety and tolerability of Deferoxamine Mesylate in patients with acute intracerebral hemorrhage Stroke 2011 42 3067 3074 10.1161/STROKEAHA.111.617589 21868742 PMC3202043

[B20] Bornkamp B Bretz F Dmitrienko A Enas G Gaydos B Hsu C Konig F Krams M Liu Q Neuenschwander B Parke T Pinheiro J Roy A Sax R Shen F Innovative approaches for designing and analyzing adaptive dose-ranging trials J Biopharm Stat 2007 17 965 995 10.1080/10543400701643848 18027208

[B21] Berry DA Mueller P Grieve AP Smith M Gatsonis C, Kass RE, Carlin B, Carriquiry A, Gelman A, Verdinelli I, West M Bayesian designs for dose-ranging drug trials Case studies in Bayesian statistics, Vol. 5 2002 New York: Springer 99 181

[B22] Krams M Lees KR Hacke W Grieve AP Orgogozo J Ford GA ASTIN: an adaptive dose -response study of UK-279,276 in acute ischemic stroke Stroke 2003 34 2543 2549 10.1161/01.STR.0000092527.33910.89 14563972

[B23] Jennison C Turnbull BW Group Sequential Methods 2000 Boca Raton: Chapman & Hall/CRC

[B24] Zhang L Rosenburger W Hinkelmann K Adaptive randomization in clinical trials Design and Analysis of Experiments, Special Designs and Applications. Volume 3 2012 Hoboken: John Wiley & Sons 251 282

[B25] Rosenberger WF Sverdlov O Hu F Adaptive Randomization for Clinical Trials J Biopharm Stat 2012 22 719 736 10.1080/10543406.2012.676535 22651111

[B26] Rosenberger WF Sverdlov O Handling covariates in the design of clinical trials Stat Sci 2008 23 404 419 10.1214/08-STS269

[B27] Antognini AB Zagoraiou M The covariate-adaptive biased coin design for balancing clinical trials in the presence of prognostic factors Biometrika 2011 98 519 535 10.1093/biomet/asr021

[B28] Jensen RK Leboeuf-Yde C Wedderkopp N Sorensen JS Minniche C Rest versus exercise as treatment for patients with low back pain and Modic changes. A randomized controlled trial BMC Med 2012 10 22 35 10.1186/1741-7015-10-22 22376791 PMC3348080

[B29] Bartlett RH Roloff DW Cornell RG Andrews AF Dillon PW Zwischenberger JB Extracorporeal circulation in neonatal respiratory failure: a prospective randomized study Pediatrics 1985 76 479 487 3900904

[B30] Eitner F Ackermann D Hilgers RD Floege J Supportive versus immunosuppressive therapy of progressive IgA Nephropathy (STOP) IgAN trial: rationale and study protocol J Nephrol 2008 21 284 289 18587715

[B31] Fiore LD Brophy M Ferguson RE D’Avolio L Hermos JA Lew RA Doros G Conrad CH O’Neil JA Jr Sabin TP Kaufman J Swartz SL Lawler E Liang MH Gaziano JM Lavori PW A point-of-care clinical trial comparing insulin administered using a sliding scale versus a weight-based regimen Clin Trials 2011 8 183 195 10.1177/1740774511398368 21478329 PMC3195898

[B32] Yuan Y Huang X Liu S A Bayesian response-adaptive covariate-balanced randomization design with application to a leukemia clinical trial Stat Med 2011 30 1218 1229 10.1002/sim.4218 21432894 PMC3086983

[B33] Fardipour P Littman G Burns DD Dragalin V Padmanabhan SK Parke T Perevozskaya I Reinold K Sharma A Krams M Planning and executing response-adaptive learn-phase clinical trials: 1. The process Drug Inf J 2009 43 713 723 10.1177/009286150904300609

[B34] Gu X Lee JJ A simulation study for comparing testing statistics in response-adaptive randomization BMC Med Res Methodol 2010 10 48 62 10.1186/1471-2288-10-48 20525382 PMC2911470

[B35] Wang SJ The bias issue under the complete null with response adaptive randomization: Commentary on “Adaptive and model-based dose-ranging trials: Quantitative evaluation and recommendation” Stat Biopharm Res 2012 2 458 461

[B36] Korn EL Freidlin B Outcome-adaptive randomization: is it useful? J Clin Oncol 2011 29 771 776 21172882 10.1200/JCO.2010.31.1423 PMC3056658

[B37] Temple R Enrichment of clinical study populations Clin Pharmacol Ther 2010 88 774 778 10.1038/clpt.2010.233 20944560

[B38] Freidlin B Simon R Evaluation of randomized discontinuation design J Clin Oncol 2005 23 5094 5098 10.1200/JCO.2005.02.520 15983399

[B39] Wang SJ Hung HMJ O’Neill RT Adaptive patient enrichment designs in therapeutic trials Biometrical J 2009 51 358 374 10.1002/bimj.200900003 19358222

[B40] Van der Baan FH Knol MJ Klungel OH Egberts ACG Grobbee DE Roes KCB Potential of adaptive clinical trial designs in pharmacogenetic research Pharmacogenomics 2012 13 571 578 10.2217/pgs.12.10 22462749

[B41] Ho TW Pearlman E Lewis D Hamalainen M Connor K Michelson D Zhang Y Assaid C Mozley LH Strickler N Bachman R Mahoney E Lines C Hewitt DJ Efficacy and tolerability of rizatriptan in pediatric migraineurs: Results from a randomized, double-blind, placebo-controlled trial using a novel adaptive enrichment design Cephalalgia 2012 32 760 765 10.1177/0333102412451358 22711898

[B42] Proschan MA Sample size re-estimation in clinical trials Biometrical J 2009 51 348 357 10.1002/bimj.200800266 19358221

[B43] Cui L Hung HMJ Wang S Modification of sample size in group sequential clinical trials Biometrics 1999 55 853 857 10.1111/j.0006-341X.1999.00853.x 11315017

[B44] Tsiatis AA Mehta C On the inefficiency of the adaptive design for monitoring clinical trials Biometrika 2003 90 367 378 10.1093/biomet/90.2.367

[B45] Jennison C Turnbull BW Adaptive and nonadaptive group sequential tests Stat Med 2006 25 917 932 10.1002/sim.2251 16220524

[B46] Mehta C Pocock SJ Adaptive increase in sample size when interim results are promising: A practical guide with examples Stat Med 2011 30 3267 3284 10.1002/sim.4102 22105690

[B47] Wittes J Brittain E The role of internal pilot studies in increasing the efficiency of clinical trials Stat Med 1990 9 65 72 10.1002/sim.4780090113 2345839

[B48] Proschan MA Two-stage sample size re-estimation based on a nuisance parameter: a review J Biopharm Stat 2005 15 559 574 10.1081/BIP-200062852 16022163

[B49] Friede T Kieser M Sample size recalculation in internal pilot study designs: a review Biometrical J 2006 4 537 555 10.1002/bimj.200510238 16972704

[B50] Kieser M Friede T Re-calculating the sample size in internal pilot study designs with control of the type I error rate Stat Med 2000 19 901 911 10.1002/(SICI)1097-0258(20000415)19:7<901::AID-SIM405>3.0.CO;2-L 10750058

[B51] Coffey CS Muller KE Controlling test size while gaining the benefits of an internal pilot design Biometrics 2001 57 625 631 10.1111/j.0006-341X.2001.00625.x 11414593

[B52] Coffey CS Kairalla JA Muller KE Practical methods for bounding type I error rate with an internal pilot design Comm Stat Theory Methods 2007 36 2143 2157 10.1080/03610920601143634 PMC3867302 24363489

[B53] Gould AL Shih W Sample size re-estimation without unblinding for normally distributed outcomes with unknown variance Comm Stat Theory Methods 1992 21 2833 2853 10.1080/03610929208830947

[B54] Friede T Kieser M Blinded sample size recalculation for clinical trials with normal data and baseline adjusted analysis Pharm Stat 2011 10 8 13 10.1002/pst.398 19943322

[B55] Maca J Bhattacharya S Dragalin V Gallo P Krams M Adaptive seamless phase II/III designs: background operational aspects and examples Drug Inf J 2006 40 463 473

[B56] Stallard N Todd S Seamless phase II/III designs Stat Methods Med Res 2010 20 623 634 20724313 10.1177/0962280210379035

[B57] Korn EL Freidlin B Abrams JS Halabi S Design issues in randomized phase II/III trials J Clin Oncol 2012 30 667 671 10.1200/JCO.2011.38.5732 22271475 PMC3295562

[B58] Conroy T Desseigne F Ychou M Bouche O Guimbaud R Becouarn Y Adenis A Raoul J Gourgou-Bourgade S Fouchardiere C Bennouna J Bachet J Khemissa-Akouz F Pere-Verge D Delbaldo C Assenat E Chauffert B Michel R Montot-Grillot C Ducreux M FOLFIRINOX versus Gemcitabine for metastatic pancreatic cancer N Engl J Med 2011 364 1817 1825 10.1056/NEJMoa1011923 21561347

[B59] Kaufmann P Thompson JLP Levy G Buchsbaum R Shefner J Krivickas LS Katz J Rollins Y Barohn RJ Jackson CE Tiryaki E Lomen-Hoerth C Armon C Tandan R Rudnicki SA Rezania K Sufit R Pestronk A Novella SP Heiman-Patterson T Kasarskis EJ Pioro EP Montes J Arbing R Vecchio D Barsdorf A Mitsumoto H Levin B Phase II trial of CoQ10 for ALS finds insufficient evidence to justify phase III Ann Neurol 2009 66 235 244 10.1002/ana.21743 19743457 PMC2854625

[B60] Levin B Ravina B, Cummings J, McDermott M, Poole RM Selection and Futility Designs Clinical Trials in Neurology: Design, Conduct, & Analysis 2012 Cambridge: Cambridge University Press 78 90

[B61] Ravina B Palesch Y The phase II futility clinical trial design Prog Neurother Neuropsych 2007 2 27 38

[B62] Levy G Kaufmann P Buchsbaum R Montes J Barsdorf A Arbing R Battista V Zhou X Mitsumoto H Levin B Thompson JLP A two-stage design for a phase II clinical trial of coenzyme Q10 in ALS Neurology 2006 66 660 663 10.1212/01.wnl.0000201182.60750.66 16534103

[B63] Tsiatis AA Information based monitoring of clinical trials Stat Med 2006 25 3236 3244 10.1002/sim.2625 16927248

[B64] Kairalla JA Muller KE Coffey CS Combining an internal pilot with an interim analysis for single degree of freedom tests Comm Stat Theory Methods 2010 39 3717 3738 10.1080/03610920903353709 PMC2965034 21037942

[B65] Kairalla JA Coffey CS Muller KE Achieving the benefits of both an internal pilot and interim analysis in large and small samples JSM Proceedings 2010 5239 5252

[B66] Tunis SR Benner J McClellan M Comparative effectiveness research: Policy context, methods development and research infrastructure Stat Med 2010 29 1963 1976 10.1002/sim.3818 20564311

[B67] Gurka MJ Coffey CS Gurka KK Internal pilots for observational studies Biometrical J 2010 5 590 603 10.1002/bimj.201000050 20857422

[B68] Scientific Advances in Adaptive Clinical Trial Designs Workshop Planning Committee Scientific Advances in Adaptive Clinical Trial Designs Workshop Summary 2010 www.palladianpartners.com/adaptivedesigns/summary

[B69] Accelerating Drug and Device Evaluation through Innovative Clinical Trial Design http://www2.med.umich.edu/prmc/media/newsroom/details.cfm?ID=1753

[B70] Neurological Emergencies Treatment Trials http://www.nett.umich.edu

[B71] The Lancet Neurology NeuroNEXT: accelerating drug development in neurology Lancet Neurol 2012 11 119 10.1016/S1474-4422(12)70008-X 22265207

[B72] Vandemeulebroeke M Group sequential and adaptive designs-a review of basic concepts and points of discussion Biometrical J 2008 50 541 557 10.1002/bimj.200710436 18663761

